# A comparative analysis of nutrition-related assessment criteria and associated nutrition performance scores of food companies across three prominent corporate sustainability assessment tools

**DOI:** 10.1017/S1368980023002215

**Published:** 2023-12

**Authors:** Ella Robinson, Jasmine Chan, Meghan O’Hearn, Dariush Mozaffarian, Gary Sacks

**Affiliations:** 1 Global Centre for Preventive Health and Nutrition, Institute for Health Transformation, Deakin University, Melbourne 3125, Australia; 2 Mozaffarian Research Group, Friedman School of Nutrition Science and Policy, Tufts University, Boston, MA, USA

**Keywords:** Corporate sustainability assessment, ESG, Food company, Nutrition, Responsible investment

## Abstract

**Objective:**

Corporate sustainability assessment tools are increasingly used to evaluate company performance on environmental, social and governance (ESG) criteria. Given the growing burden of diet-related disease and nutrition-related business risks, it is important to understand the scope of nutrition-related ESG data currently available. This study aimed to compare the nutrition-related assessment criteria and associated food company performance across three prominent assessment tools.

**Design:**

Key attributes and assessment criteria of two civil society-led and one commercially available corporate sustainability assessment tools were extracted and compared for the year 2021. Company performance scores for twenty-five major food and beverage manufacturers using these three tools were analysed by nutrition domain: ‘Product Portfolio’, ‘Labelling’, ‘Marketing’, ‘Accessibility and Affordability’, ‘Governance and Reporting’, ‘Stakeholder Engagement’ and ‘Employee Health’. To enable comparison between tools, company performance scores were assigned to categories of *low* (score = 0–25 % score or D), *moderately low* (25–50 % or C), *moderately high* (50–75 % or B) and *high* (75–100 % or A).

**Setting:**

Global.

**Participants:**

N/A.

**Results:**

The tools covered similar nutrition domains; however, there was heterogeneity in the assessment criteria used to evaluate each domain. When applied to assess the performance of twenty-five major food and beverage manufacturers, a median nutrition-related performance score of *moderately low* or *low* was observed across all tools. The highest scoring domain was ‘Governance and Reporting’, and the lowest scoring domains were ‘Product Portfolio’ and ‘Accessibility and Affordability’.

**Conclusions:**

Greater standardisation of the nutrition-related criteria against which food companies are assessed is needed as part of efforts to drive improvements in food company practices.

Dietary risk factors, including undernutrition and overnutrition, are the leading cause of mortality and morbidity globally and are a major contributor to diet-related non-communicable diseases^(1)^. A key driver of unhealthy diets is food systems that are dominated by unhealthy ultra-processed foods and beverages that are heavily promoted and widely accessible^(2)^. Within such food systems, large, multinational food and beverage manufacturers have built up increasing power and influence^(3,4)^. In order to improve health outcomes, food systems transformation that enables the provision of healthy, accessible, and affordable foods and beverages to all populations is required^(5)^. As part of efforts to transform food systems and improve population diets, it is critical that large food and beverage manufacturers are held to account for their role^(6)^.

Across a range of industries and sectors, efforts to promote better corporate governance and provide data on corporate practices have led to a rapid growth in corporate sustainability assessment tools^(7,8)^. Measuring and comparing corporations using criteria-led metrics, for example through the use of ratings or benchmarking, has been used extensively to assess the performance of companies across various environmental, social and governance (ESG) issues^(8–10)^. A broad range of stakeholders, including governments, civil society and the financial sector, are increasingly demanding comparable ESG data to inform decision-making and advocacy efforts. These types of assessments can contribute to efforts to hold food companies accountable for their role in improving population diets^(11,12)^.

As stewards of capital, investors can influence corporate governance and corporate accountability^(13)^, including for food industry corporations^(14–16)^. Through their role as shareholders and lenders, institutional investors use ESG data to understand, track and evaluate sustainability performance, as well as to meet client demands and ethical considerations^(17)^. Importantly, ESG data can be used to form the basis of engagement and voting decisions that may impact corporate practices^(18,19)^. Institutional investors primarily obtain ESG data on corporate performance from commercially available research and ratings providers such as MSCI, ISS ESG, Sustainalytics, Bloomberg, Thomson Reuters and RobecoSAM^(20,21)^. Corporate sustainability assessments are also conducted by civil society groups. In the area of nutrition, the most prominent corporate sustainability assessments include those coordinated by the Access to Nutrition Initiative (ATNI), the World Benchmarking Alliance (WBA), the Food Foundation and INFORMAS (International Network for Food and Obesity/NCDs Research, Monitoring and Action Support). All of these tools assess various sectors of the food and beverage industry on their performance in relation to aspects of nutrition, with particular focus areas (e.g. undernutrition and overnutrition) varying depending on the tool^(11,22–24)^.

There is a large body of work examining how ESG metrics and methodologies differ across corporate sustainability assessment tools (including ESG ratings)^(7,25–27)^ and how corporations are measured and perform in relation to different ESG criteria and ESG ratings^(8,21)^. The differences in methodologies across tools, and subsequent variability in ESG data and performance assessment scores assigned to companies, have drawn major criticism from the investment sector^(20,27,28)^. Previous research has found that several of the most widely used sustainability reporting frameworks, including the Sustainability Accounting Standards Board (SASB) and the Global Reporting Initiative (GRI) Sector Disclosures, include some nutrition-related topics and reporting metrics for relevant sectors^(14)^. There is also emerging recognition that metrics for nutrition should be systematically and comprehensively included within the ESG framework to facilitate adequate uptake and use of nutrition-related data within institutional investment decision-making^(14,16,29)^. However, to our knowledge, no academic research has reviewed the nutrition-specific methodologies and performance ratings of food companies across different corporate sustainability assessment tools.

This study aimed to: (1) compare and contrast nutrition-related assessment criteria across prominent corporate sustainability assessment tools and (2) compare the nutrition-related performance scores of twenty-five major food companies across the selected tools. The goal of the analysis was to work towards consensus on performance metrics and disclosure requirements and greater consistency in nutrition-related ESG data.

## Methods

### Data selection

We set out to compare nutrition-related company assessments from a range of relevant tools, including prominent civil society-led and commercially available corporate sustainability assessment tools.

#### Civil society-led corporate sustainability assessment tools

We identified prominent nutrition-related civil society-led corporate sustainability assessment tools that evaluate the food industry based on our previous research and knowledge of the field^(11,14,16,30)^, supplemented by a targeted internet search of the grey literature in November–December 2021. Information about the methodology of each tool (e.g. from relevant documents and websites) was scanned to identify key characteristics of each tool and determine their suitability to be included in the comparative analysis. A list of tools initially identified and key characteristics of these tools are outlined in the online supplementary material, Supplemental Table S1. Tools were selected for inclusion in this study if they included nutrition-related assessment topics, incorporating a focus on overnutrition, included an assessment of food and beverage manufacturers, assessed companies at the global level, were active as of 2021, and made their assessment results publicly available in English. Tools were excluded if they assessed companies at the country/national level only. The two tools that were selected for inclusion were the ATNI ‘Global Index’ 2021 and the WBA ‘Food and Agricultural Benchmark’ 2021.

#### Commercially available corporate sustainability assessment tool

Data from ISS ESG (2021), one of the largest ESG ratings providers, was selected for inclusion in this study, informed by discussions with industry contacts as part of previous studies^(15,31)^. These discussions had indicated that ISS ESG data were well regarded in terms of data quality and were widely used by the responsible investment community in a range of countries. The ISS ESG ‘Corporate Rating’ includes data on nutrition-related topics (amongst a wide range of other ESG-related topics) and covers all major listed global food and beverage companies^(32)^. Due to the commercial nature of the data from the ESG rating providers and budget limitations, we were only in a position to include data from one ESG rating provider as part of this study.

### Data extraction

#### Nutrition-related assessment criteria across tools

The most recent assessment reports and methodology documents for the two civil society-led tools were downloaded from their websites in November–December 2021. The ISS ESG dataset was accessed through an online portal in May 2021.

Metrics across tools were classified as relevant if they mentioned terms related to nutrition, healthy/unhealthy food or healthy/unhealthy diets, and/or were directly related to topic areas highlighted as critical for improving population diets in established frameworks (e.g. key characteristics of food environments defined by INFORMAS, including food composition, food labelling, food marketing, food provision, food retail, food prices, food trade and investment^(33)^). For these purposes, ‘nutrition’ was conceptualised to refer to either undernutrition (i.e. related to stunting, wasting, underweight and micronutrient deficiencies) or overnutrition (including overweight and obesity). Other metrics were excluded from the analysis. For example, while the ISS ESG ‘Corporate Rating’ had several metrics within its ‘Corporate governance and business ethics’ domain, these were excluded because they did not specifically mention governance related to nutrition. The only exceptions were indicators related to lobbying and political engagement, which for WBA and ISS ESG did not specifically mention nutrition. These indicators were included because they were seen as being highly relevant to nutrition-related corporate political activity, a commonly identified mechanism by which food companies exert their influence^(4)^. Food-related metrics that were related to environmental topics, social welfare topics (such as human rights in the food supply chain), food waste and food safety topics were excluded because they were beyond the scope of our analysis.

Overarching characteristics of each tool were extracted, such as the number of companies assessed, the sources of data used by the tool, and the year and jurisdiction in which the tool was implemented. We also extracted relevant nutrition-related assessment criteria from each tool, including nutrition-related assessment topics, indicators and weightings. Nutrition-related assessment criteria were then classified according to six key nutrition domains. These domains were chosen based on WHO and other public health-recommended actions for the private sector to address population diets^(11,34)^. These included ‘Product Portfolio’, ‘Labelling’, ‘Marketing’, ‘Governance and Reporting’, ‘Accessibility and Affordability’ and ‘Stakeholder Engagement’. An additional domain, ‘Employee Health’, was included for comparison purposes because this domain was assessed by two of the tools. Therefore, a total of seven domains were classified for comparison across tools.

#### Company performance scores across tools

To investigate how performance scores of food companies compared across the three tools when applied in practice, a sample of twenty-five of the largest food and beverage manufacturers globally were chosen for comparison across the three tools. For this analysis, we chose to focus on large transnational food and beverage manufacturers because they were the only food sector assessed by all three tools (ATNI ‘Global Index’ tool, the WBA ‘Food and Agricultural Benchmark’ and the ISS ESG ‘Corporate Rating’), were almost all publicly listed companies (and therefore had company assessment data available for download as part of the ISS ESG ‘Corporate Rating’) and have been shown to hold substantial corporate power and influence within global food systems^(3,4)^. Data on overall ESG performance (if applicable), overall nutrition performance and nutrition-domain-specific performance scores of each food and beverage manufacturer were extracted from the three datasets.

The ISS ESG dataset included quantitative performance scores for each company across the various ESG metrics assessed, as well as composite ESG scores by topic (e.g. overall ‘social’ score) and an overall ESG rating. For each of the twenty-five companies, a detailed qualitative data report was downloaded (download date: 14 May 2021). For several companies, their ownership structure was either private or co-operatively held, and as such, no report was available for download from the ISS ESG online data portal and performance scores for these companies were noted as not applicable (NA).

All three tools used different scoring scales to assess company performance. To allow for comparison of food company performance ratings across the tools, scoring was converted to a percentile out of 100 and assigned a colour-coded performance rating of *low* (0–25 % score or D−, D, D+ rating), *moderately low* (25–50 % or C−, C, C+), *moderately high* (50–75 % or B−, B, B+) and *high* (75–100 % or A−, A, A+). Refer to Table 1 for a scoring scale comparison across the tools.

**Table 1:**
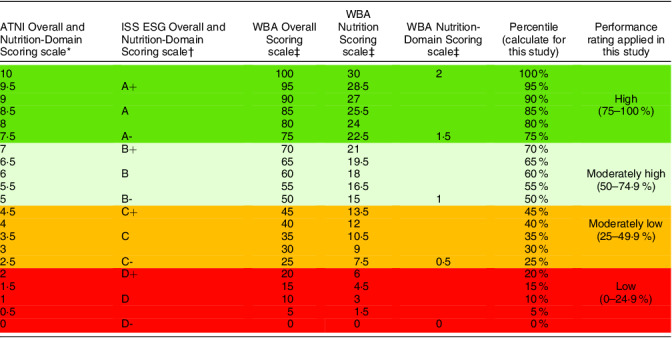
Scoring scale comparison and performance rating applied (in this study) for overall and nutrition-domain scores across the Access to Nutrition Initiative ‘Global Index’ (ATNI), World Benchmarking Alliance ‘Food and Agricultural Benchmark’ (WBA) and the ISS ESG ‘Corporate Rating’ (ISS ESG)

ATNI, Access to Nutrition Initiative; ISS, Institutional Shareholder Services; ESG, environmental, social and governance; WBA, World Benchmarking Alliance.

*ATNI ‘Global Index’ used a numeric scale of 1–10 for overall performance and nutrition-domain-level performance.

†ISS ESG ‘Corporate Rating’ used an alphabetical scale from D- to A+ for overall performance and nutrition-domain-level performance.

‡WBA ‘Food and Agricultural Benchmark’ used a numeric scale from 1 to 100 for overall performance, 1 to 30 for nutrition overall performance and a numeric scale of 0 to 2·0 for nutrition-domain-level performance.

Colour code: Dark green = High (75–100%); Light green = Moderately high (50–74.9%); Yellow = Moderately low (25–49.9%); Red = Low (0%–24.9%).

### Findings

#### Comparison of key attributes across tools

Of the tools included, only the ATNI ‘Global Index’ focused solely on evaluating nutrition performance (i.e. 100 % of the total score was attributed to nutrition). For the WBA ‘Food and Agricultural Benchmark’, topics directly related to nutrition made up 30 % of the overall company performance score, with other topics related to Governance and Strategy (10 %), Environment (30 %) and Social inclusion (30 %). Within the ISS ESG ‘Corporate Rating’, food companies were assessed on nutrition-related criteria under the Society and Product Responsibility topic. The overall weighting for this domain was 24 %; however, after excluding metrics not directly related to nutrition (such as human rights, food safety and animal welfare), approximately 15·4 % of the overall company performance score was attributed to nutrition. For ISS ESG, the metrics related to nutrition were not able to be summed into a total nutrition score. As such, for ISS ESG, this score was based on one metric called Health and Nutrition (within the Society and Product Responsibility topic) which covered domains on ‘Labelling’, ‘Product Portfolio’ and ‘Governance and Reporting’. Details on key attributes including relevant food industries evaluated and assessment criteria used across the three corporate sustainability assessment tools are outlined in Table 2.

**Table 2:** Key attributes across two civil society-led and one commercially available corporate sustainability assessment tools

Organisation	Tool	Year	Description	Food industries included	Number of packaged food and beverage manufacturers included	Data sources	Nutrition-related assessment topics	Number of indicators/scoring criteria	Weighting*	Nutrition-related domain classification for this study
Access to Nutrition Initiative (ATNI)	Global Index	2021	The Access to Nutrition Initiative (ATNI) through its flagship ‘Global Index’, assesses the largest global food and beverage manufacturers on their nutrition and undernutrition-related policies, practices and product portfolios^(22)^.	• Food and beverage manufacturers	25 companies	• Public data • Extensive consultation with companies	Products	34	35 %	‘Product Portfolio’
							Labelling	18	10 %	‘Labelling’
							Marketing	27	20 %	‘Marketing’
							Accessibility	14	15 %	‘Accessibility and Affordability’
							Governance	24	12·5 %	‘Governance and Reporting’
							Engagement	15	5 %	‘Stakeholder Engagement’
							Lifestyles	18	2·5 %	‘Employee Health’
World Benchmarking Alliance (WBA)	Food and Agricultural Benchmark	2021	The World Benchmarking Alliance (WBA) is a major non-profit benchmarking organisation that assesses influential companies on their contribution to the Sustainable Development Goals. The WBA ‘Food and Agricultural Benchmark’ assesses the largest food and agricultural companies on their commitments across four key domains: Environment, Governance and Strategy, Social Inclusion and nutrition^(24)^.	• Food and beverage manufacturers/processors • Food retailers • Restaurants • Food service	233 companies (food and beverage manufacturers/processors)	• Public data • Surveys with companies	Availability of healthy foods† Clear and transparent labelling† Responsible marketing† Accessibility and affordability of healthy foods† Sustainable development strategy§ Governance and accountability for sustainable development§ Stakeholder engagement§ Responsible lobbying and political engagement fundamentals|| Workforce nutrition†	3 3 3 3 4 2 1 2	NA‡ NA‡ NA‡ NA‡ NA‡ NA‡ NA‡	‘Product Portfolio’ ‘Labelling’ ‘Marketing’ ‘Accessibility and Affordability’ ‘Governance and Reporting’ ‘Governance and Reporting’ ‘Stakeholder Engagement’ ‘Stakeholder Engagement’ ‘Employee Health’
Institutional Shareholder Services (ISS) ESG	ESG Corporate Rating	2021	The ISS ESG ‘Corporate Rating’ provides a detailed assessment of a company’s ESG performance^(35)^.	• Restaurants • Food retail • Food distributors • Hypermarkets and supercentres • Soft drinks • Packaged foods and meat	1511 companies (soft drinks, packaged foods and meat)¶	• Public data • Dialogue with companies	Nutrition targets relating to product portfolio** Strategy to reduce critical additives in food and beverages** Social impact of products and services Nutrition labelling** Responsible marketing Position on health and nutrition aspects of products** Stakeholder dialogue Relations with governments and influence on public policy Community involvement	NA NA NA NA NA NA NA NA	1·34–2·40 % 0·67–1·20 % 4·21–6·00 % 0·67–1·20 % 1·26–2·53 % 0·67–1·20 % 0·50–0·70 % 1·50–2·10 % 0·50–0·70 %	‘Product Portfolio’ ‘Product Portfolio’ ‘Product Portfolio’ ‘Labelling’ ‘Marketing’ ‘Governance and Reporting’ ‘Governance and Reporting’ ‘Stakeholder Engagement’ ‘Stakeholder Engagement’

ESG, environmental, social and governance; NA, not applicable; NDA, non-disclosure agreement.

* For the ISS ESG ‘Corporate Rating’, weightings fluctuated depending on the company assessed. The lowest-highest weightings are reported for the sample of twenty-five food and beverage manufacturers assessed as part of this study.

† Assessed within the nutrition domain, which was weighted at 30 % of the overall performance score.

‡ NA, not applicable as information on weightings at the metric level was not found.

§ Assessed within the Governance and Reporting domain, which was weighted at 10 % of the overall performance score.

|| Assessed within the Social Inclusion domain, which was weighted at 30 % of the overall performance score.

¶ As at May 2021.

** Assessed within the Health and Nutrition domain, which was weighted at 3·36–6·00 % of the overall performance score.

#### Comparison of assessment criteria by tool

ATNI included by far the most criteria for assessing nutrition performance, with 150 total indicators in the ‘Global Index’. The WBA ‘Food and Agricultural Benchmark’ included a total of nine nutrition-related assessment topics with an associated twenty-three relevant scoring criteria under Nutrition, Governance and Strategy and Social Inclusion. The ISS ‘ESG Corporate Rating’ included six nutrition-related assessment topics, with some topics including a score or range of scores, and some including commentary only (with no score). Due to the way in which the assessment within these topics was reported, we were not able to accurately determine the number of indicators within each topic. All initiatives had at least one assessment criterion under each of the seven key nutrition domains of interest to this comparative analysis, except for the ISS ESG ‘Corporate Rating’ which did not have any criteria related to ‘Employee Health’ or ‘Accessibility and Affordability’. A comparison of the assessment criteria across tools within each nutrition domain is outlined in Table 3.

**Table 3:** Assessment criteria across tools, by nutrition-related domain

Nutrition-related domain	ATNI ‘Global Index’ 2021	WBA ‘Food and Agricultural Benchmark’ 2021	ISS ESG ‘Corporate Rating’ 2021
Product portfolio			
(Re)formulation to reduce risk nutrients/foods	✓	✓	✓
(Re)formulation to increase beneficial nutrients/foods	✓	✓	X
Product development to address priority populations	✓	X	X
Proportion or sales of healthy *v*. unhealthy products/foods in portfolio	✓	✓	✓
Independent assessment of product portfolio healthiness	✓	X	X
Definition/classification used to determine healthiness of products	✓	✓	X
Labelling			
Back-of-pack nutrition information (e.g. nutrients and serving/portion size)	✓	✓	✓
Front-of-pack labelling	✓	✓	✓
Responsible use of health and nutrition claims	✓	X	✓
Online nutrition information	✓	X	X
Marketing			
General responsible marketing commitments	✓	✓	✓
Responsible marketing to children commitments	✓	✓	✓
Marketing of breastmilk substitutes*	X†	X	✓
Marketing in/around places where children gather	✓	X	X
Sponsorship using unhealthy products/brands	✓	X	X
Promotional techniques targeted at children (toys, games and characters)	✓	X	X
Marketing budget for healthy *v*. unhealthy products	✓	✓	X
Third-party auditing and compliance	✓	X	X
Accessibility and affordability			
General commitments to improve accessibility and/or availability of healthy foods	✓	✓	X
Improving affordability of healthy foods	✓	✓	X
Governance and reporting			
Commitment/prioritisation of nutrition by company	✓	✓‡	✓
Reporting of progress against nutrition targets and objectives	✓	✓‡	✓‡
Accountability for nutrition strategy (e.g. senior level)	✓	✓‡	X§
Stakeholder engagement			
Disclosure and reporting of stakeholder relationships	✓	✓	✓
Lobbying practices	✓	✓	✓
Political contributions	✓	✓	✓
Employee health			
Strategies to improve employee nutrition (health and wellness programmes, support for breast-feeding)	✓	✓	X

* For relevant companies only.

† ATNI has a separate breastmilk substitute and complementary foods index (BMS/CF Marketing Index) that specifically rates the marketing practices of BMS/CF companies and their alignment with the WHO International Code of Marketing of Breast-milk Substitutes. This index is detailed within their 2021 Global Index methodology documents.

‡ Assessment indicator refers to sustainable development activities broadly but includes mention of nutrition/health as part of this.

§ ISS ESG has an indicator that assesses the integration of sustainability objectives into the variable remuneration of the executive management team; however, it does not include mention of nutrition/health as part of this.

#### Comparison of overall company performance by tool

The twenty-five major global food and beverage manufacturers performed relatively consistently across the ATNI ‘Global Index’, the WBA ‘Food and Agricultural Benchmark’ and the ISS ESG ‘Corporate Rating’ tools. Median performance scores equated to an overall and nutrition-related performance rating of *moderately low* across all tools. Given ATNI’s sole focus on nutrition, companies’ nutrition performance scores were equivalent to their overall performance score. Of note, no companies across any of the tools received an overall or nutrition performance rating of *high* (75–100 %). Refer to Table 4 for overall and nutrition-related performance scores. The highest, mid-range and lowest overall performing companies were similar across the three tools. For example, Nestlé, Unilever and Danone were in the top four highest performing companies across all three tools, and Tingyi, Mengniu and Yili were in the four lowest performing companies across all three tools.

**Table 4:**
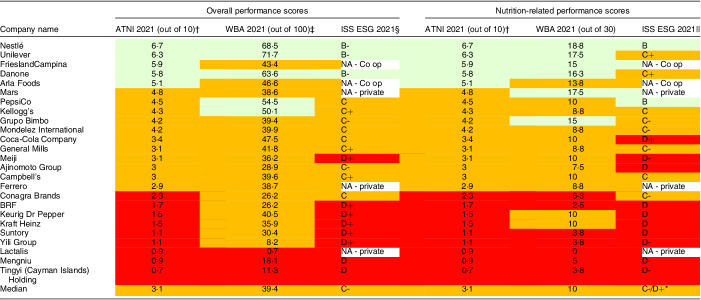
Overall and nutrition-related performance scores for twenty-five major food and beverage manufacturers in 2021 across three corporate sustainability assessment tools

ATNI, Access to Nutrition Initiative ‘Global Index’; ISS, Institutional Shareholder Services Environmental Social Governance ‘Corporate Rating’; NA, not applicable as no data was available; WBA, World Benchmarking Alliance ‘Food and Agricultural Benchmark’.

Colour code: red = *low* performance rating (0–24·9 %); orange = *moderately low* performance rating (25–49·9 %); light green = *moderately high* performance rating (50–74·9 %); dark green = *high* performance rating (75–100 %).

*The median score for ISS ESG was reported as *moderately low*.

†Nutrition metrics make up 100 % of the overall score.

‡Nutrition metrics make up 30 % of the overall score.

§Nutrition metrics (including community involvement, relations with governments and influence on public policy, stakeholder dialogue, responsible marketing, social impact of products and services, health and nutrition) make up approximately 15·40 % of the total score.

||Score is for the Health and Nutrition metric only, which makes up 3·36–6·00 % of the overall score, depending on the company assessed.

#### Comparison of assessment criteria and company performance by nutrition-related domain

Heterogeneity in the assessment criteria and company performance scores across the three tools was observed at the nutrition-related domain level (see online supplementary material, Supplemental Tables S2·1–2·7 for nutrition-domain scores).

##### Product portfolio

All tools included metrics to assess product (re)formulation to reduce risk nutrients (such as Na, sugar and fat) and energy content; however, where ATNI and ISS ESG assessed specific reformulation targets, WBA referred to (re)formulation as a ‘focus area’ under the provision of more healthy and nutritious foods. ISS ESG under a separate metric assessed the proportion of a company’s product portfolio and share of net sales that contributed to/obstructed the UN Sustainable Development Goals (SDG) (including SDG 2 combating hunger and malnutrition and SDG 3 ensuring health); however, it did not define a nutritional profiling system to assess portfolio healthiness. ATNI was the only tool that included a comprehensive independent assessment of the overall healthiness of a company’s product portfolio. For this analysis, ATNI used two internationally recognised nutrient profiling models (the Health Star Rating system and the WHO Regional Nutrient Profiling Model).

‘Product Portfolio’ was the lowest scoring across all domains assessed. Companies received a *moderately low* median score in the ATNI and WBA tools and a *low* median score in the ISS ESG. There was some variation between scores assigned to companies across tools. Only four companies in ATNI and three companies in WBA received a *low* rating, compared to more than 70 % of companies in ISS ESG. Danone, Nestlé and Unilever all scored *high* or *moderately high* in ATNI and WBA and in ISS ESG’s metric that assessed nutrient targets. However, these three companies performed *low* or *moderately low* in ISS ESG’s metric that assessed a company’s actual proportion of sales from healthy *v*. unhealthy products.

##### Labelling

All tools assessed back-of-pack and front-of-pack nutrition labelling to some degree. ATNI and WBA generally assessed company commitments and reporting, while ISS ESG assessed the extent to which companies adopted labelling across their products. ATNI assessed the responsible use of health and nutrition claims on product packaging (whether these claims were only placed on products deemed as ‘healthy’), and ISS ESG had one metric on the use of unsubstantiated health or environmental product claims within their Responsible Marketing topic.

Median scores across the three tools varied for the ‘Labelling’ domain (WBA: *moderately high*; ATNI: *moderately low*; ISS: *low*). No companies scored well on ‘Labelling’ metrics for ISS ESG, whereas some companies scored *moderately high* or *high* in the other two tools. Nestlé and Unilever scored *high* in ATNI for commitments to implement interpretive front-of-pack labelling and nutrition and health claims and *high* in WBA for committing to transparent nutritional information and front-of-pack labelling. In contrast, Nestlé and Unilever scored *moderately low* in ISS ESG, because they determined that less than 1 % of their product portfolio was labelled according to traffic light labelling or a comparable interpretive system.

##### Marketing

ATNI, WBA and ISS ESG all had metrics to assess responsible marketing commitments targeted to all consumers and children specifically. ATNI included by far the most metrics to assess companies on their policies around marketing to children and adolescents, with a number of indicators assessing the comprehensiveness and breadth of policies related to marketing to children, promotional techniques and sponsorship. ISS ESG had one metric to assess ‘sensitive products’ which looked at whether commitments aligned with the WHO International Code of Marketing of Breastmilk Substitutes (BMS) (only applicable to companies that produced BMS products). ATNI assessed BMS marketing as part of a separate index (the BMS/Complementary Foods Marketing Index). ATNI and WBA both included a metric to assess disclosure of the marketing budget spent on promoting healthy products and commitments to increasing this budget.

Company performance scores were relatively homogenous for the ‘Marketing’ domain, with an overall *moderately low* median score across tools. FrieslandCampina was the highest performing company in ATNI, but only a *moderately low* performing company in WBA, as it was awarded points by ATNI for making commitments around marketing strategies that reach priority populations and adhering to the International Chamber of Commerce (ICC) Framework for Responsible Food and Beverage Marketing Communications.

##### Accessibility and affordability

ATNI and WBA included metrics to assess general commitments related to improving the accessibility or availability of healthy products/foods as well as metrics to assess food affordability, including pricing arrangements and other commercial activities designed to improve the affordability of healthy products. Both focused on undernutrition and food insecurity amongst vulnerable groups, with metrics related to increasing healthy and affordable products in these particular settings (and a focus on addressing micronutrient deficiencies by ATNI). No comparable ‘Accessibility and Affordability’ metrics were included in ISS ESG.

‘Accessibility and Affordability’ was one of the lowest scoring domains (median company assessments were ATNI: *low;* WBA: *moderately low*; ISS ESG: not available). Only two companies (Nestlé and FrieslandCampina) in ATNI and five (Nestlé, FrieslandCampina, Danone, Unilever and Arla Foods) in the WBA scored > 50 % (*moderately high)*, demonstrating a comprehensive pricing and distribution strategy or commitment for healthier products and products targeted to certain groups. Arla Foods’ efforts to increase the accessibility and affordability of healthy foods in low-income markets led to a *high* rating in WBA, while it was scored *moderately low* in ATNI because its strategy did not extend to rural, middle- and high-income markets.

##### Governance and reporting

ATNI included metrics that assessed nutrition-related governance (e.g. the extent to which nutrition was embedded within the corporate strategy, reporting against nutrition targets and objectives, formal accountability systems for nutrition goals). In contrast, WBA and ISS ESG assessed governance in relation to sustainable development more broadly. WBA included metrics to assess the integration of sustainable development targets and objectives within the company’s corporate strategy and governance and accountability for this strategy. ISS ESG included metrics to assess the scope, quality, level of assurance and coverage of broadly defined sustainability reporting. Neither tool focused specifically on nutrition governance and reporting, but nutrition (or customer and product responsibility in the case of ISS ESG) was included as one of the potential topic areas.

‘Governance and Reporting’ was the highest overall median scoring domain (ATNI: *moderately low*; WBA: *moderately high* for governance and accountability and *high* for sustainable development strategy; ISS ESG: *moderately low*). WBA had the highest number of *moderately high* and *high* scoring companies in this domain. Like other domains, differences in company scores were observed across tools. For example, Suntory scored *high* in WBA for targets and reporting associated with its sustainability strategy, but *low* in ATNI for limited disclosure, SMART (specific, measurable, achievable, relevant, time-bound) targets and formalised reporting of nutrition policies, and *moderately low* in ISS ESG for poor-quality sustainability reporting in terms of accuracy, regularity, comparability and assurance.

##### Stakeholder engagement

ATNI assessed commitments, disclosure and evidence of nutrition-related stakeholder engagement, funding for philanthropic, nutrition education/active lifestyle programmes and external research activities as well as nutrition-related corporate political activities (e.g. lobbying and political donations). In contrast, WBA assessed disclosure and reporting of stakeholder engagement activities in relation to sustainable development issues more broadly, with responsible lobbying and political engagement covered under the Social Inclusion topic, but not specific to nutrition. ISS ESG also included metrics to assess overarching corporate political activity practices not specific to nutrition (including political contributions and lobbying) as well as community involvement (community programmes run through a foundation or in cooperation with external organisations).

Companies received a median score of *moderately low* or *low* in all tools for lobbying and political engagement metrics; however, they scored *moderately high* for stakeholder engagement (WBA) and community involvement (ISS ESG). WBA awarded *high* scores for twelve companies’ stakeholder engagement activities; however, these companies all performed poorly on lobbying and political engagement activities. No companies scored *high* in ATNI, which found limited disclosure of nutrition-related lobbying and stakeholder engagement activities. Almost all companies assessed by ISS ESG, except Unilever, Nestlé and Danone, scored *low* for a lack of disclosure around payments to or from governments, political contributions, and lobbying expenditures.

##### Employee health

ATNI and WBA included metrics related to ‘Employee Health’, while ISS ESG did not. ATNI assessed the scope and impact of employee health and wellness support and community-supporting healthy eating and active lifestyle programmes provided by the company, as well as breast-feeding support for mothers, while WBA only included a small number of metrics to assess the provision of healthy foods in the workplace, nutrition education and breast-feeding support.

Overall, companies received *moderately low* median scores across both tools. However, variation in the indicators assessed by each tool meant that the performance scores of individual companies were not directly comparable.

## Discussion

This study found that three prominent corporate sustainability assessment tools that assess food companies on nutrition performance assessed similar nutrition-related domains. ATNI’s ‘Global Index’ included the most comprehensive set of nutrition-related assessment criteria, when compared to those tools that assessed companies on ESG topics more generally (WBA’s ‘Food and Agricultural Benchmark’ and the commercially available ISS ESG ‘Corporate Rating’). When these tools were applied to twenty-five of the largest global food and beverage manufacturers, the overall (i.e. across all aspects included in the respective tool) and nutrition-related performance ratings assigned to companies were generally consistent. Companies received an overall and nutrition-related median performance rating of *moderately low* across all tools. No companies received a performance rating of *high* across any of the tools. Across tools and companies, performance was highest in ‘Governance and Reporting’, and lowest in ‘Product Portfolio’ and ‘Accessibility and Affordability’, with heterogeneity in domain-specific scoring across companies.

Whilst the broad nutrition topics included within the three tools were similar, this study found that assessment criteria across tools were not standardised. Multiple instances were identified where individual companies were assigned different performance ratings across the three tools due to variations in assessment criteria. The lack of consistent data on the ESG performance of companies has previously been noted as a key challenge for investors when integrating ESG considerations as part of investment decision-making^(36,37)^. Large-scale ESG rating providers all use different methodologies to assess the ESG performance of food companies, and the overall performance ratings of some food companies can vary^(20,28)^. Furthermore, there are an increasing number of civil society-led benchmarking tools publishing data on corporate nutrition performance across the food value chain^(22–24,30,38)^. Variability in the metrics, weightings and subjectivity of scoring systems across tools, including those highlighted in this analysis, limits the comparability and consistency of nutrition-related food company performance data and is likely to impact the way in which stakeholders, like investors, interpret this data.

As well as inconsistencies in assessment criteria, the variation in overall performance scores across large food and beverage manufacturers and their scores in the ‘Disclosure and Reporting’ domain would suggest that food company sustainability reporting is widely inconsistent. There was also variability in the way that sustainability reporting was assessed for quality and breadth across the tools, for example, companies assessed in WBA received a median performance rating of *high* for sustainability reporting, but in ISS ESG they received a median performance rating of *moderately low*. All three tools primarily relied on publicly available sustainability reporting to conduct their assessments, which is voluntarily disclosed, supplemented by company engagement. Previous research has shown that mandatory ESG disclosure requirements increase the availability and quality of ESG reporting^(39)^, and various stakeholders in the financial sector are now calling for alignment between regulators and standard setters to create globally consistent and comparable corporate ESG disclosure requirements^(40)^.

The findings from this study suggest that nutrition-related governance and the provision of healthier products are not necessarily correlated. The lowest scoring domains in this study were ‘Product Portfolio’ and ‘Accessibility and Affordability’, while the ‘Governance and Reporting’ domain received the highest median score in ATNI and WBA and second highest in ISS ESG. For example, Nestlé and Unilever were two of the highest performing companies in the ‘Governance and Reporting’ domain across all three tools, but only 29 % and 18 % of their product portfolio were assessed as being ‘healthy’ according to the Health Star Rating nutrient profile model^(22)^. ATNI was the only tool to provide a comprehensive assessment of the healthiness of a company’s product portfolio as part of the ‘Product Portfolio’ domain, using internationally recognised nutrient profiling models to do so (the Australian government endorsed Health Star Rating system and the WHO Regional Nutrient Profiling Model). Whilst corporate nutrition governance, disclosure and reporting are important for internal and external accountability arrangements, without improvements to the healthiness of a company’s product portfolio, there is unlikely to be significant improvements to population diets and health outcomes. This finding suggests that different weightings for each nutrition domain warrant close consideration, with an argument for product portfolio assessment metrics to receive a higher weighting compared to other metrics. Furthermore, it will be important for corporate sustainability assessment tools to assess product portfolio healthiness using government-endorsed classification systems that reflect the latest public health evidence, with increasing attention to the use of ‘food-based’ (rather than ‘nutrient-based’) classification systems and systems that take into account the level of food processing (e.g. the NOVA classification system^(41)^).

### Implications for future research, policymakers and investors

The findings from this study point to the need for standardisation of methodologies across food company sustainability assessment tools. The WBA and the Food Foundation report that they are working towards standardised methodology to assess the food and agriculture sector on topics related to nutrition, environment and social inclusion^(42)^, indicating promising progress to standardise methods across civil society-led initiatives in future. Furthermore, a programme of work in the USA is exploring the development of harmonised, evidence-based ESG-Nutrition metrics to guide food sector practices towards nutrition, health and equity^(29)^. This type of research will be crucial for ensuring nutrition-related assessment metrics for the food sector are appropriately developed and weighted to measure companies on aspects of their practices that are likely to have the most impact on population health. Importantly, previous studies indicate that nutrition as an ESG issue is still an emergent area of interest for investors^(14,16,43)^. Groups such as ATNI are working with investors to ensure better uptake of nutrition-related ESG data^(44)^; however, it has been argued that more concerted efforts are needed to raise the prominence of nutrition-related business risks and associated financial implications, with a particular focus on engagement with the financial sector^(19,31,45)^.

This study also identified clear inconsistencies in the depth and quality of reporting by food companies on nutrition-related issues, which impacts corporate sustainability assessments and associated ESG performance ratings. As noted by the Food Foundation, mandatory nutrition-related reporting requirements for food companies will help to ensure more consistent information is provided to end user stakeholders, including investors, policymakers and civil society^(46)^. There are several jurisdictions and organisations working to develop globally agreed sustainability reporting requirements^(40)^; however, the extent to which nutrition-related reporting will be considered as part of such efforts is unclear. Encouragingly, in 2022, the UK government committed to explore mandatory reporting requirements for large food businesses in relation to food waste and sales-based food production metrics (healthy, unhealthy and animal products) as part of their National Food Strategy^(47)^. However, the UK government recently announced its intention to rather explore voluntary approaches in this area^(48)^. Critically, in order for reporting metrics to be effective at driving corporate accountability for population nutrition, they must be evidence-based, developed in collaboration with public health nutrition experts, and include strong monitoring and compliance mechanisms. Future research should explore the extent to which nutrition-related reporting requirements will be included within global sustainability reporting directives, if adopted.

An important finding from this study was that, despite differences in assessment criteria across the three corporate sustainability assessment tools, the performance ratings of food companies were similar. On the one hand, this may imply that corporate sustainability assessments that focus on a small selection of nutrition metrics, such as those included in ISS ESG, could potentially perform effectively as a proxy for a more comprehensive set of nutrition metrics, such as those included in ATNI. However, the usefulness of these datasets will be dependent on the context in which it is being used by end user stakeholders, including NGOs, policymakers, regulators and investors. As an example, for investors, if nutrition-related performance ratings are used to inform portfolio selection (e.g. to screen food companies in or out of a fund depending on minimum standards of performance), then it is likely that a smaller subset (‘proxy’) metrics may prove adequate. However, if investors are using nutrition-related performance ratings as part of active ownership activities, that is, active participation in corporate engagement, voting and shareholder resolutions^(49)^, then detailed nutrition-domain data will likely be critical to inform these activities. Active ownership has been the primary focus of investor engagement initiatives conducted by groups like ATNI which outline investor asks of food companies related to nutrition^(19)^. In working towards consensus on nutrition-related performance metrics and disclosure requirements, it will be important to understand and take into account the ways in which data will be used by different end users and in different contexts.

### Strengths and limitations

To our knowledge, this is the first study to compare nutrition-related assessment criteria and performance ratings assigned to food companies across different corporate sustainability assessment tools. We included two civil society-led tools and one commercial dataset to compare how methodologies and nutrition performance ratings differ across major companies in the food industry and thus the scope of data that may be available to end user stakeholders like investors. We also assessed how the performance scores of twenty-five global food and beverage manufacturers differed across the three tools when applied in practice. This approach highlighted practical examples of how differences in assessment criteria can result in inconsistent food company performance scores. Limitations should also be considered. Due to resource constraints, only one commercially available ESG dataset was purchased and used as part of the comparative analysis. ISS ESG was chosen based on previous research that identified it as one of the most in-depth corporate sustainability assessment tools likely to have nutrition metrics. Nevertheless, the ISS ‘ESG Corporate Rating’ dataset is unlikely to be representative of the broader ESG research and data analytics market. This limits the conclusions that can be drawn around the scope of commercially available, nutrition-related ESG data within the investment sector. Furthermore, several other international and national civil society-led benchmarking tools exist that were not included as part of this study due to these not meeting the inclusion criteria for this study. These tools also provide data on food company performance across various sectors of the food industry, for example, the BIA-Obesity tool (international), Plating Up Progress (UK) and the ATNI ‘Food Retailer Index’ (UK). Future studies should explore a wider breadth of ESG data ratings and civil society-led benchmarking tools to better understand the scope of commercially and publicly available nutrition-related data. Further research could additionally focus on assessment tools relevant to other subsectors across the food system value chain, such as retail, restaurants, processors and agricultural producers. Moreover, whilst this study comparatively analysed assessment criteria and company performance across key domains and topic areas in which authoritative bodies have recommended nutrition-related actions from food companies to improve population diets, we did not provide comprehensive recommendations on what nutrition-related metrics should be prioritised for assessment based on their likely population nutrition impact. Future research should explore the nutrition-related metrics for food companies that are likely to be the most meaningful for relevant stakeholders and the most impactful for addressing population nutrition goals. Lastly, tools and performance data were extracted for 2021 only. Given the dynamic nature of ESG reporting, this may not be representative of the metrics and methodologies used previously and into the future.

### Conclusion

There are an increasing number of corporate sustainability assessment tools that evaluate the nutrition performance of food companies. This study shows that, while the performance of food companies across three prominent corporate sustainability assessment tools was similar, assessment criteria are heterogenous. Standardisation and prioritisation of nutrition assessment criteria across corporate sustainability assessment tools are needed to drive changes in company nutrition practices and consistent use of nutrition-related ESG data by end user stakeholders, including investors and government policymakers.

## Supporting information

Robinson et al. supplementary material 1 Robinson et al. supplementary material

Robinson et al. supplementary material 2 Robinson et al. supplementary material

## Supplementary material

For supplementary material accompanying this paper visit https://doi.org/10.1017/S1368980023002215
