# Supplementary material for: A comparative analysis of nutrition-related assessment criteria and associated nutrition performance scores of food companies across three prominent corporate sustainability assessment tools
Source: Public Health Nutr. 2023 Oct 23;26(12):2677–90. doi: 10.1017/S1368980023002215 (PMC10755394; doi:10.1017/S1368980023002215)
Supplement: Robinson et al. supplementary material 1 — Robinson et al. supplementary material [file S1368980023002215sup001.docx]

***Supplementary material***

***Table S1: Civil-society led corporate sustainability assessment tools initially identified for this study and key attributes across tools***

| **Organisation** | **Tool** | **Year** | **Jurisdiction** | **Description** | **Relevant food industries assessed** | **Number of companies** | **Assessment topics** | **Data sources** |
| --- | --- | --- | --- | --- | --- | --- | --- | --- |
| **Access to Nutrition Initiative (ATNI)** | **BMS/CF Index** | 2021 | Global | The Access to Nutrition Initiative (ATNI) Breast-milk Substitute/Commercial Formula (BMS/CF) Index assesses the largest breast-milk substitute and commercial formula companies on the extent to which their marketing practices align with WHO guidance. | - Breast-milk substitute and commercial formula companies | 9 | Alignment with WHO Code for BMS/CF policy, management systems and disclosure; BMS/CF marketing practices in low or middle-income countries | - Public data - Extensive consultation with companies |
| **Access to Nutrition Initiative (ATNI)** | **Global Index** | 2021 | Global | The ATNI through its flagship ‘Global Index’, assesses the largest global food and beverage manufacturers on their nutrition and undernutrition related policies, practices and product portfolios. | - Food and beverage manufacturers | 25 | Governance; Products; Accessibility; Marketing; Lifestyles; Labeling; Engagement. | - Public data - Extensive consultation with companies |
| **Access to Nutrition Initiative (ATNI)** | **India Spotlight Index** | 2020 | India | The ATNI India Spotlight Index assesses the largest food and beverage manufacturers on their nutrition and undernutrition related commitments, practices and disclosure. | - Food and beverage manufacturers | 16 | Governance, Accessibility, Marketing, Employee and Consumer Lifestyles, Labeling and Engagement | - Public data - Extensive consultation with companies |
| **Access to Nutrition Initiative (ATNI)** | **UK Retailer Index** | 2021 | United Kingdom | In 2021, ATNI released its methodology for assessing food retailers as part of a new ‘UK Retailer Index’. This Index follows on from a 2020 UK ‘spotlight’ food retailer benchmarking initiative conducted by ATNI, and assesses food retailers on their disclosure related to nutrition, diets and health. | - Food retailers | 11 | Governance; Nutrient profiling model; Product formulation; In-store promotion, pricing and distribution; Media and on-pack advertising; Accessibility of nutrition information and labelling; Engagement with stakeholders and policy makers; Infant and young child nutrition | - Public data - Extensive consultation with companies |
| **Access to Nutrition Initiative (ATNI)** | **U.S Index** | 2018 | United States | The ATNI U.S Index assessed the largest food and beverage manufacturers in the United States on their malnutrition-related commitments, practices and disclosure. | - Food and beverage manufacturers | 11 | Governance; Products; Accessibility; Marketing; Lifestyle; Labeling; Engagement; Marketing of breast-milk substitutes | - Public data - Extensive consultation with companies |
| **World Benchmarking Alliance** | **Food and Agricultural Benchmark** | 2021 | Global | The World Benchmarking Alliance (WBA) is a major non-profit benchmarking organization that assesses influential companies on their contribution to the Sustainable Development Goals. The WBA ‘Food and Agricultural Benchmark’ assesses the largest food and agricultural companies on their commitments across four key domains: environment, governance and strategy, social inclusion and nutrition. | - Food and beverage manufacturers/   processors   - Food retailers - Restaurants - Food service | 255 food sector companies (of 350 total across agriculture and food) | Environment; governance and strategy; social inclusion; **nutrition** (Availability of healthy foods; Accessibility and affordability of healthy foods; Clear and transparent labelling; Responsible marketing; Workforce nutrition; Food safety) | - Public data - Surveys with companies |
| **The Food Foundation** | **Plating up Progress** | **2021** | United Kingdom | The Food Foundation ‘Plating Up Progress’ initiative assesses and measures food industry performance across 10 major topics related to healthy and sustainable diets, environment and social inclusion. | - Supermarkets - Quick service restaurants - Contract caterers - Food service - Casual dining - Restaurants - Wholesalers | 29 | Environment; social inclusion; **healthy and sustainable diets** (Sales of healthy foods; procurement/sales from fruit and vegetables and animal versus plant-based proteins; nutrition labelling; marketing strategy; accessibility and affordability of healthy foods). | - Public data - Opportunity for fact checking by companies |
| **INFORMAS** (the International Network for Food and Obesity/NCDs Research, Monitoring and Action Support) | **BIA-Obesity Australia** | **2018** | Australia | The BIA-Obesity tool and process was developed to benchmark the nutrition‐related policies, commitments and practices of food companies at a national level. In Australia, BIA-Obesity was implemented in 2018 to assess the largest Australian food companies on their nutrition-related policies and commitments. | - Food retailers - Quick Service Restaurants - Food and beverage manufacturers | 34 | Corporate strategy; Product formulation; Nutrition labelling; Promotion practices; Product accessibility; Relationships with external groups. | - Public data - Consultation with companies |
| **INFORMAS** | **BIA-Obesity Canada** | **2019** | Canada | The BIA-Obesity Canada assessed the largest food and beverage manufacturers on their nutrition‐related policies and commitments. | - Food and beverage manufacturers | 22 | Corporate strategy; Product formulation; Nutrition labelling; Promotion to children and adolescents; Product accessibility; Relationships with external organisations. | - Public data - Consultation with companies |
| **INFORMAS** | **BIA-Obesity New Zealand** | **2018** | New Zealand | The BIA-Obesity New Zealand assessed the largest food and beverage manufacturers, food retailers and quick service restaurants on their nutrition‐related policies and commitments. | - Food retailers - Quick Service Restaurants - Food and beverage manufacturers | 25 | Corporate population nutrition strategy; Product formulation; Nutrition labelling; Product and brand promotion; Product accessibility; Relationships with other organisations. | - Public data - Consultation with companies |
| **INFORMAS** | **BIA-Obesity Malaysia** | **2019** | Malaysia | The BIA-Obesity Malaysia assessed the nutrition‐related policies and commitments of food companies across three sectors, as well as market surveys of company products. | - Food retailers - Quick Service Restaurants - Food and beverage manufacturers | 33 | Corporate strategy; Product formulation; Nutrition labelling; Promotion practices; Product accessibility; Relationships with external groups. | - Public data - Market surveys - Consultation with companies |
| **INFORMAS** | **BIA-Obesity Thailand** | **2019** | Thailand | The BIA-Obesity Thailand conducted a publicly available policy assessment of major food companies nutrition-related policies and commitments. | - Food retailers - Quick Service Restaurants - Food and beverage manufacturers | 19 | - Corporate strategy; Product formulation; Nutrition labelling; Promotion practices; Product accessibility; Relationships with external groups. | - Public data only |

ATNI = Access to Nutrition Initiative; BIA-Obesity = Business Impact Assessment – Obesity and population nutrition; BMS = Breast-Milk Substitute; CF = Commercial Formula; INFORMAS = International Network for Food and Obesity / Non-communicable Diseases (NCDs) Research, Monitoring and Action Support; WBA = World Benchmarking Alliance.
