# Supplementary material for: A comparative analysis of nutrition-related assessment criteria and associated nutrition performance scores of food companies across three prominent corporate sustainability assessment tools
Source: Public Health Nutr. 2023 Oct 23;26(12):2677–90. doi: 10.1017/S1368980023002215 (PMC10755394; doi:10.1017/S1368980023002215)
Supplement: Robinson et al. supplementary material 2 — Robinson et al. supplementary material [file S1368980023002215sup002.docx]

***Supplementary material***

***Tables S2.1-S2.7 Nutrition-related domain scores***

**Table S2.1 Product portfolio**

| **Company name** | **ATNI  (Products)** | **WBA (Availability of healthy foods)** | **ISS ESG (Nutrition targets relating to product portfolio)** | **ISS ESG (Strategy to reduce critical additives in foods and beverages)** | **ISS ESG (Social impact of products and services)** |
| --- | --- | --- | --- | --- | --- |
| Danone | 7.2 | 2 | A- | D- | C+ |
| Nestlé | 7.1 | 1 | A- | B | D+ |
| FrieslandCampina | 6.8 | 2 | NA - Co op | NA - Co op | NA - Co op |
| Unilever | 5.7 | 2 | B- | D- | C- |
| Mars | 5.5 | 2 | NA - private | NA - private | NA - private |
| Arla Foods | 5.4 | 1.5 | NA - Co op | NA - Co op | NA - Co op |
| Grupo Bimbo | 5 | 2 | D- | D- | D+ |
| Mondelez International | 4.5 | 0.5 | D- | B+ | D- |
| PepsiCo | 4.1 | 1 | A+ | D- | D |
| Yili Group | 4 | 0 | D- | D- | B- |
| Meiji | 3.9 | 0.5 | D- | D- | C- |
| General Mills | 3.8 | 0.5 | D | C+ | D+ |
| Ferrero | 3.5 | 0.5 | NA - private | NA - private | NA - private |
| Campbell's | 3.4 | 1 | D- | A | D+ |
| Kellogg's | 3.4 | 0.5 | D- | A | D+ |
| Conagra Brands | 3.4 | 0.5 | D- | C+ | D+ |
| Mengniu | 3.3 | 0.5 | D- | D- | C+ |
| Kraft Heinz | 3.3 | 2 | D- | D+ | D+ |
| Ajinomoto Group | 2.6 | 0.5 | D- | D- | C- |
| Suntory | 2.6 | 0.5 | D+ | D+ | D+ |
| BRF | 2.5 | 0 | D | D- | D+ |
| Coca-Cola Company | 2.4 | 0.5 | D+ | D- | D+ |
| Lactalis | 2.4 | 0 | NA - private | NA - private | NA - private |
| Keurig Dr Pepper | 1.6 | 0.5 | D- | D+ | D |
| Tingyi (Cayman Islands) Holding | 1.4 | 0.5 | D- | D- | D |
|  |  |  |  |  |  |
| Median scores | 3.5 | 0.5 | D- | D- | D+ |

**Table S2.2 Labelling**

| **Company name** | **ATNI  (Labelling)** | **WBA (Clear and transparent labelling)** | **ISS ESG (Nutrition labelling)** |
| --- | --- | --- | --- |
| Unilever | 8.5 | 1.5 | C+ |
| Nestlé | 8.4 | 1.5 | C- |
| Mondelez International | 7 | 1 | D+ |
| FrieslandCampina | 6.7 | 1 | NA - Co op |
| Danone | 6.4 | 1.5 | D+ |
| Arla Foods | 6.2 | 1 | NA - Co op |
| PepsiCo | 6.1 | 1 | C |
| Kellogg's | 5.9 | 1 | C |
| Mars | 5.5 | 1.5 | NA - private |
| Coca-Cola Company | 5.1 | 1 | D+ |
| Campbell's | 4.2 | 1 | C+ |
| Grupo Bimbo | 3.9 | 1 | C- |
| General Mills | 3.3 | 1 | D+ |
| Meiji | 2.7 | 1 | D- |
| Ferrero | 2.4 | 1 | NA - private |
| Conagra Brands | 1.8 | 0 | C+ |
| Ajinomoto Group | 1.7 | 0.5 | D- |
| Kraft Heinz | 1.5 | 0 | C |
| BRF | 0.7 | 0.5 | D- |
| Keurig Dr Pepper | 0.7 | 1 | C+ |
| Lactalis | 0.1 | 0 | NA - private |
| Suntory | 0.1 | 0 | D- |
| Mengnui | 0 | 0 | D- |
| Tingyi (Cayman Islands) Holding | 0 | 0 | D- |
| Yili Group | 0 | 0 | D- |
|  |  |  |  |
| Median scores | 3.3 | 1 | D+ |

**Table S2.3 Marketing**

| **Company name** | **ATNI  (Marketing)** | **WBA (Responsible marketing)** | **ISS ESG (Responsible marketing)** |
| --- | --- | --- | --- |
| FrieslandCampina | 7.9 | 0.5 | NA - Co op |
| Mars | 7.4 | 0.5 | NA - private |
| Nestlé | 7.3 | 0.5 | B |
| Unilever | 6.8 | 1 | A- |
| Danone | 6.5 | 0.5 | B |
| Ferrero | 5 | 0.5 | NA - private |
| Mondelez International | 5 | 0.5 | B |
| Coca-Cola Company | 4.8 | 0.5 | C |
| Kellogg's | 4.4 | 0.5 | B |
| PepsiCo | 4.4 | 0.5 | A+ |
| Arla Foods | 4.2 | 0.5 | NA - Co op |
| Campbell's | 3.7 | 0.5 | C |
| General Mills | 3.6 | 0.5 | C- |
| Keurig Dr Pepper | 3.1 | 0.5 | C- |
| Grupo Bimbo | 3 | 0.5 | D |
| Kraft Heinz | 2.5 | 0.5 | C+ |
| Meiji | 2.4 | 0.5 | D |
| Ajinomoto Group | 2.2 | 0.5 | D- |
| Conagra Brands | 2.1 | 0.5 | D |
| BRF | 0.4 | 0 | D- |
| Lactalis | 0 | 0 | NA - private |
| Mengnui | 0 | 0.5 | D- |
| Suntory | 0 | 0 | D- |
| Tingyi (Cayman Islands) Holding | 0 | 0 | D |
| Yili Group | 0 | 0 | D- |
|  |  |  |  |
| Median scores | 3.6 | 0.5 | C- |

**Table S2.4 Accessibility and affordability**

| **Company name** | **ATNI (Accessibility)** | **WBA  (Accessibility and affordability of healthy foods)** | **ISS ESG** |  |
| --- | --- | --- | --- | --- |
| FrieslandCampina | 7.7 | 1.5 | NA- no equivalent domain |  |
| Nestlé | 6 | 1 | NA- no equivalent domain |  |
| Danone | 4.8 | 1 | NA- no equivalent domain |  |
| Unilever | 4.6 | 1 | NA- no equivalent domain |  |
| Arla Foods | 4 | 2 | NA- no equivalent domain |  |
| Kellogg's | 4 | 0 | NA- no equivalent domain |  |
| Grupo Bimbo | 3.6 | 1 | NA- no equivalent domain |  |
| PepsiCo | 3.2 | 0 | NA- no equivalent domain |  |
| Ajinomoto Group | 1.8 | 0.5 | NA- no equivalent domain |  |
| Mars | 1.8 | 0 | NA- no equivalent domain |  |
| Coca-Cola Company | 1.7 | 0.5 | NA- no equivalent domain |  |
| Meiji | 1.7 | 1 | NA- no equivalent domain |  |
| General Mills | 0.3 | 0.5 | NA- no equivalent domain |  |
| BRF | 0.2 | 0 | NA- no equivalent domain |  |
| Campbell's | 0.2 | 0.5 | NA- no equivalent domain |  |
| Conagra Brands | 0.2 | 0 | NA- no equivalent domain |  |
| Kraft Heinz | 0.2 | 0 | NA- no equivalent domain |  |
| Mondelez International | 0.2 | 0 | NA- no equivalent domain |  |
| Yili Group | 0.2 | 0.5 | NA- no equivalent domain |  |
| Keurig Dr Pepper | 0.1 | 0 | NA- no equivalent domain |  |
| Ferrero | 0 | 0 | NA- no equivalent domain |  |
| Lactalis | 0 | 0 | NA- no equivalent domain |  |
| Mengnui | 0 | 0.5 | NA- no equivalent domain |  |
| Suntory | 0 | 0 | NA- no equivalent domain |  |
| Tingyi (Cayman Islands) Holding | 0 | 0 | NA- no equivalent domain |  |
|  |  |  |  |  |
| Median score | 0.3 | 0.5 |  |  |

**Table S2.5 Governance and reporting**

| **Company name** | **ATNI (Governance)** | **WBA  (Governance and accountability for sustainable development)** | **WBA (Sustainable development strategy)** | **ISS ESG (Stakeholder dialogue)** | **ISS ESG (Position on health and nutrition aspects of products)** |
| --- | --- | --- | --- | --- | --- |
| Nestlé | 9.7 | 1.5 | 1.5 | A- | A- |
| FrieslandCampina | 8.2 | 1 | 2 | NA - Co op | NA - Co op |
| Unilever | 8.1 | 2 | 2 | A+ | A- |
| Danone | 7.1 | 1.5 | 2 | A- | A- |
| Grupo Bimbo | 6.4 | 1 | 0.5 | C+ | A+ |
| Ajinomoto Group | 6.3 | 0.5 | 1 | C+ | B- |
| PepsiCo | 6.2 | 1.5 | 2 | B | A- |
| Kellogg's | 6.2 | 1 | 2 | B | B- |
| Arla Foods | 5.8 | 1 | 2 | NA - Co op | NA - Co op |
| Mondelez International | 5.2 | 1.5 | 2 | C+ | B+ |
| Coca-Cola Company | 4.9 | 1 | 2 | C+ | C+ |
| Campbell's | 4.6 | 1 | 1.5 | B | A- |
| Meiji | 4.2 | 1 | 2 | C+ | C |
| Mars | 4.2 | 1.5 | 0.5 | NA - private | NA - private |
| BRF | 3.9 | 1.5 | 1.5 | C- | C |
| General Mills | 3.8 | 1 | 1.5 | A- | C+ |
| Kraft Heinz | 3.4 | 1 | 2 | C+ | D+ |
| Mengniu | 3.4 | 1 | 1 | C+ | D+ |
| Ferrero | 3.3 | 1 | 1.5 | NA - private | NA - private |
| Conagra Brands | 3.1 | 1 | 0.5 | C+ | C+ |
| Yili Group | 2.3 | 0.5 | 0 | C- | D- |
| Keurig Dr Pepper | 1.3 | 1 | 0.5 | C+ | D |
| Tingyi (Cayman Islands) Holding | 1.3 | 1 | 1 | D | D- |
| Suntory | 0.8 | 1 | 2 | C- | D |
| Lactalis | 0.1 | 0 | 0 | NA - private | NA - private |
|  |  |  |  |  |  |
| Median score | 4.2 | 1 | 1.5 | C+ | C+ |

**Table S2.6 Stakeholder engagement**

| **Company name** | **ATNI (Engagement)** | **WBA (Stakeholder engagement)** | **WBA (lobbying and political engagement fundamentals)** | **ISS ESG (Relations with governments and influence on public policy)** | **ISS ESG (Community involvement)** |
| --- | --- | --- | --- | --- | --- |
| Nestlé | 6.6 | 1.5 | 0.5 | C- | B |
| FrieslandCampina | 5.9 | 2 | 0.5 | NA - Co op | NA - Co op |
| Unilever | 5.7 | 2 | 0.5 | C+ | A+ |
| Danone | 5.3 | 1.5 | 0.5 | C- | A+ |
| Kellogg's | 4.9 | 1.5 | 0.5 | D- | C+ |
| PepsiCo | 4.7 | 2 | 0.5 | D+ | B- |
| Ajinomoto Group | 4 | 1 | 0 | D+ | C |
| General Mills | 3.6 | 1.5 | 0 | D+ | A |
| Coca-Cola Company | 3.2 | 1.5 | 0.5 | D+ | C+ |
| Mondelez International | 3.2 | 0 | 0 | D | A+ |
| Arla Foods | 3.1 | 1 | 0.5 | NA - Co op | NA - Co op |
| Mars | 2.6 | 0 | 0.5 | NA - private | NA - private |
| Kraft Heinz | 2.5 | 1.5 | 0.5 | D | B |
| Grupo Bimbo | 2.3 | 0 | 0.5 | D- | C |
| Meiji | 2.2 | 0 | 0 | D | D+ |
| Ferrero | 2.1 | 0.5 | 0.5 | NA - private | NA - private |
| Campbell's | 2 | 1.5 | 0.5 | D+ | B+ |
| Conagra Brands | 2 | 0 | 0 | D+ | A |
| BRF | 1.8 | 0.5 | 0.5 | D | B- |
| Tingyi (Cayman Islands) Holding | 1 | 0.5 | 0 | D- | C |
| Keurig Dr Pepper | 0.9 | 1.5 | 0 | D+ | C+ |
| Suntory | 0.9 | 1.5 | 0 | D- | C+ |
| Mengniu | 0.6 | 0.5 | 0.5 | D- | C- |
| Yili Group | 0.3 | 0 | 0 | D- | D+ |
| Lactalis | 0 | 0 | 0 | NA - private | NA - private |
|  |  |  |  |  |  |
| Median score | 2.5 | 1 | 0.5 | D+ | C+ to B- |

**Table S2.7 Employee health**

| **Company name** | **ATNI (Lifestyles)** | **WBA (Workforce nutrition)** | **ISS ESG** |
| --- | --- | --- | --- |
| Unilever | 7.6 | 0.5 | NA- no equivalent domain |
| Nestlé | 7.3 | 2 | NA- no equivalent domain |
| Danone | 5.1 | 1 | NA- no equivalent domain |
| PepsiCo | 4.3 | 0.5 | NA- no equivalent domain |
| Ferrero | 4.1 | 0.5 | NA- no equivalent domain |
| Mondelez International | 4.1 | 0 | NA- no equivalent domain |
| Arla Foods | 3.9 | 0 | NA- no equivalent domain |
| FrieslandCampina | 3.7 | 0.5 | NA- no equivalent domain |
| Ajinomoto Group | 3.4 | 0.5 | NA- no equivalent domain |
| Grupo Bimbo | 3.3 | 1 | NA- no equivalent domain |
| Mars | 3.3 | 2 | NA- no equivalent domain |
| General Mills | 2.5 | 0.5 | NA- no equivalent domain |
| Kellogg's | 2.5 | 1 | NA- no equivalent domain |
| Tingyi (Cayman Islands) Holding | 2 | 0 | NA- no equivalent domain |
| Kraft Heinz | 1.8 | 0.5 | NA- no equivalent domain |
| Meiji | 1.8 | 0.5 | NA- no equivalent domain |
| Coca-Cola Company | 1.4 | 0.5 | NA- no equivalent domain |
| Suntory | 1.4 | 0.5 | NA- no equivalent domain |
| Keurig Dr Pepper | 1.3 | 0.5 | NA- no equivalent domain |
| BRF | 1.2 | 0 | NA- no equivalent domain |
| Campbell's | 0.7 | 0 | NA- no equivalent domain |
| Conagra Brands | 0.7 | 0 | NA- no equivalent domain |
| Yili Group | 0.3 | 0 | NA- no equivalent domain |
| Lactalis | 0 | 0 | NA- no equivalent domain |
| Mengniu | 0 | 0 | NA- no equivalent domain |
|  |  |  |  |
| Median score | 2.5 | 0.5 |  |

| **Percentile** | **Performance rating** |
| --- | --- |
| 100% |  |
|  |  |
|  | High |
|  | (75%-100%) |
|  |  |
| 75% |  |
|  |  |
|  |  |
|  | Moderately high |
|  | (50%-74.9%) |
| 50% |  |
|  |  |
|  | Moderately low |
|  | (25%-49.9%) |
|  |  |
| 25% |  |
|  |  |
|  | Low |
|  | (0%-24.9%) |
|  |  |
| 0% |  |

**Performance rating color code**
